# What we (still don’t) know about wood-degrading fungi: an overview of threats and utilization potentials

**DOI:** 10.1007/s00253-026-14043-1

**Published:** 2026-09-28

**Authors:** Elisabeth Tamayo, Felix Bachmann, Tian Cheng, Tim K. Felle, Marcello Nussbaumer, J. Philipp Benz

**Affiliations:** Professorship of Fungal Biotechnology in Wood Science, Holzforschung München, TUM School of Life Sciences, Technical University of Munich, Freising, Germany

**Keywords:** Wood-degrading fungi, Wood degradation, Macroscopic treats, Molecular mechanisms, Meta-analysis, Utilization potentials

## Abstract

Wood, being one of the most abundant materials on Earth, is a hierarchically structured lignocellulosic biocomposite that has evolved to resist decomposition by other organisms. While lignin is thought to have evolved in the Devonian (~ 400–390 Ma), leading to the deposition of vast coal reserves in the Carboniferous (~ 360–299 Ma), a diverse group of fungi evolved around that time to precisely break through this barrier and use it as carbon source. The emerging “wood-degrading fungi” since then play a fundamental role in global nutrient cycling, carbon flow, and material turnover. The significance of wood-degrading fungi lies both in their applicability thanks to their enzyme systems, transporters, and metabolites, and in their being threats to forests, wood products, and health. This duality is similar, albeit maybe less known to the public, than the one in the food industry, where fungi have both useful applications (e.g., in food coloring, food fermentation, and as protein sources) and risks (e.g., through mycotoxins). Moreover, studies on fungal colonization and substrate attack in connection with fungal degradation in the built environment have expanded our mechanistic understanding of their natural behavior. In the present mini-review, we provide an overview of the mechanisms by which fungi degrade wood and discuss both the threats to living trees and wood products associated with wood-degrading fungi as well as their potential for added value in the bioeconomy. We furthermore aim to highlight important macroscopic and microscopic aspects to provide an integrative view of the mechanisms involved, including data retrieved by genomic and literature meta-analyses. This mini-review integrates insights from the literature with exploratory analyses of diverse databases to provide a holistic view of wood-degrading fungi, supporting future perspectives and a multidimensional understanding of these organisms.

## Colonization of wood: how fungal decay begins

The colonization of wood by decay fungi can occur through the germination of spores at susceptible areas such as debarked timber or wounds in living trees, which can be caused by abiotic (mechanical damage, weather, etc.) or biotic (birds, insects, etc.) factors, or by access through the soil with their mycelium (Schmidt 2010; Shortle and Dudzik 2012). Spores and sclerotia are able to survive in the soil for years and germinate when encountering suitable conditions, including nutrients on a root surface (Carlile et al. 2001). Some roots even attract hyphae, which subsequently enter through wounds or young roots, spread in the cortex, and finally enter the xylem after invading the endodermis, stele, and phloem tissue (Carlile et al. 2001). Wood-degrading fungi can spread on wood quite fast if the conditions are suitable. Depending on the fungal and tree species as well as the environmental conditions, deterioration of mechanical properties can be observed already after 2 weeks (Cartwright et al. 1931; Mulholland 1954; Wilcox 1978). Apart from brown, white, and soft rot fungi (see for rot types below), many other microorganisms such as bacteria and yeasts colonize the wood during degradation (Blanchette and Shaw 1978). Different competitive interactions shape the local community, which will ultimately determine the degradation rate and type (Hiscox et al. 2015; Fukasawa 2021). These antagonisms include the exchange of volatiles, hyphal interference, and mycoparasitism (Boddy 2000).

## Macro- and microscopic traits of fungal wood degradation

Wood-degrading fungi are either specialized on colonizing living trees (obligatory parasites and endophytes) or dead wood (obligatory saprotrophs) or can facultatively do both (Schmidt 2010). After the death of a tree, parasites often become saprotrophs, and the wood becomes prone to a larger variety of saprotrophic fungi, going through various stages (Boddy 2001; Schmidt 2010). Endophytes—fungi that can live within healthy plant tissues without causing any kind of obvious symptoms—likely participate in degradation processes after felling (Cline et al. 2018). For indoor wood, *Serpula lacrymans* is an especially problematic species and notorious in Europe due to its ability to spread fast across and through surfaces that do not provide nutrition or water (Watkinson and Eastwood 2012). This is possible due to the translocation of resources through mycelial cords (Lindahl and Olsson 2004). Such a “guerilla” growth strategy focusing on exploration exhibits substantially less interconnectivity by branching and anastomosis of the hyphal network than the exploitative “phalanx” strategy that is favored in nutrient-rich substrates (Aguilar-Trigueros et al. 2022). The same strategy helps parasites like *Armillaria mellea* overcome the defense mechanisms of trees in the form of rhizomorphs (Carlile et al. 2001). They differ from mycelial cords in their root-like growth behavior driven by apical regions of densely packed hyphae, whereas mycelial cords form further away from the growth front by young hyphae fusing with already existing ones (Yafetto 2018).

**Fig. 1:**
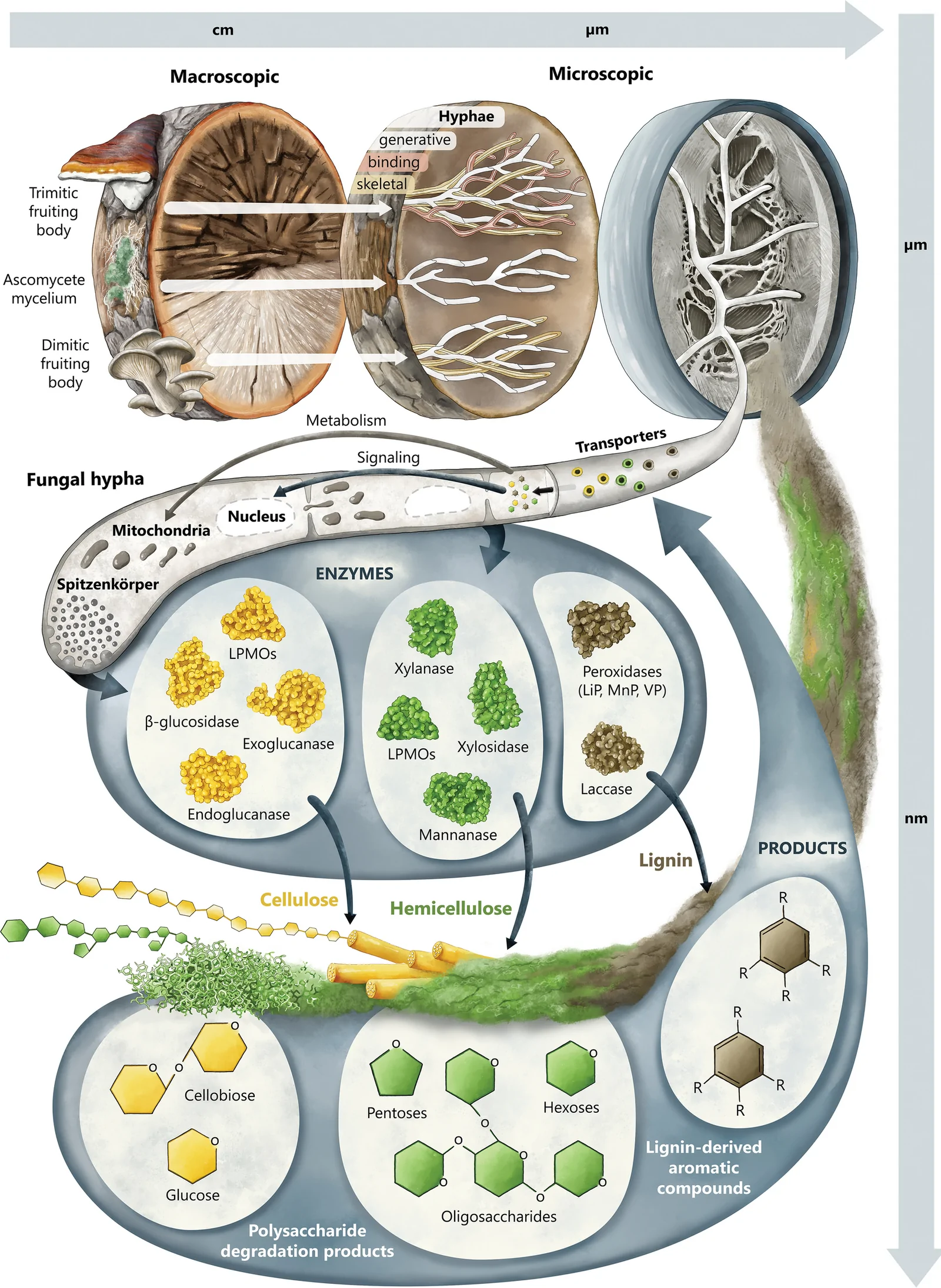
Fungal wood degradation from macro- to nano-scale. At the macroscopic level, three representative fungi with different hyphal types are shown: *Fomitopsis pinicola*, a brown rot fungus with trimitic fruiting bodies (generative, binding, and skeletal hyphae), *Trichoderma* sp., an Ascomycete fungus forming monomitic fruiting bodies (with generative hyphae only), and *Pleurotus ostreatus*, a white rot fungus that develops a dimitic fruiting body (with skeletal and generative hyphae). The differences in wood appearance resulting from the different rot types (brownish and cube-shaped for *F. pinicola* and whitish-fibrous for *P. ostreatus*) are indicated. At the microscopic level, the various hyphal types are indicated in their combinations and an electron-microscopic-like view into a wood cell with hyphae degrading the plant cell wall surface. Lower part of the figure: Fungi feed by osmotrophic nutrient uptake. A single generative hypha is shown that is actively secreting lignocellulolytic enzymes into a representative cell wall fragment, consisting of cellulose (yellow), hemicellulose (green), and lignin (brown). The secreted enzymes are colored correspondingly. By the activity of the enzymes, soluble/mobile degradation products are released (glucose and cellobiose from cellulose; pentoses, hexoses, and oligosaccharides from hemicellulose, and aromatic/phenolic compounds from lignin), which will diffuse back to the hypha and are taken up by corresponding transporters. Within the hypha, the compounds are both metabolized and act as inducers, regulating gene expression in the nuclei by triggering specific signaling cascades. This will result in increased protein production and secretion across the hyphal tip and the septa

After building up sufficient biomass, fungi can reproduce through spores often released from a fruiting body under suitable conditions (Sakamoto 2018). Specific morphological traits of the fruiting bodies, such as shape, size, colors, and features like pores or gills facilitate classification, but even more important are microscopic aspects like spore appearance and hyphal types (Corner 1932; Prasher 2015; Wei et al. 2022). While different morphologies of spores all serve the optimization of reproduction through dispersion over space and time, hyphae fulfill different functions based on their structure (Wyatt et al. 2013; Porter and Naleway 2022). Based on the branching and cell wall thickness, hyphae are categorized into generative, skeletal, or binding hyphae (Fig. 1) (Donk 1964; Prasher 2015). The so-called monomitic system comprises only generative hyphae, whereas di- and trimitic systems also contain one or both of the other types (Donk 1964). With increasing variety of hyphal types, the consistency of fruiting bodies gets tougher due to the strong mechanical properties of skeletal and binding hyphae (Prasher 2015; Porter and Naleway 2022). This system is based on the hyphae found in fruiting bodies, and some literature also indicates that skeletal and binding hyphae are unique for them and not present in the vegetative mycelium (Corner 1932; Donk 1964; Prasher 2015). Donk (1964) and Prasher (2015) describe that in the initial stages of fruiting, only generative hyphae are present and from them, the other two types emerge in later stages. However, for some wood-degrading fungi, di- and trimitic systems have also been observed in the vegetative mycelium (Buchalo et al. 2009; Balaeş and Tănase 2012a, b; Petre and Tanase 2013a, b; Mazian et al. 2023). The growth stage in which they appear is most likely species-specific, as Buchalo et al. (2009) found skeletal and binding hyphae in the vegetative mycelium of only one fungus, whereas five of the inspected species’ fruiting bodies were described as trimitic and four as dimitic (Buchalo et al. 2009). Notably, a recent study found that even the generative hyphae in the vegetative mycelium of one investigated dimitic (*Grifola frondosa*) and one trimitic (*Ganoderma sichuanense*) species have thicker cell walls and higher stiffness than the ones of a monomitic species (*Sparassis spathulata)* (Gallagher et al. 2025). With few exceptions (Kiss et al. 2019; Carrillo et al. 2020), the genetic influence on hyphal morphology or even basic structure is not yet understood.

## Wood-decay types: white rot, brown rot, and soft rot

As already mentioned in the previous section, three main wood-rot types are classically distinguished based on the appearance of the degraded wood, i.e., white rot, brown rot, and soft rot (Hartig 1878; Schwarze et al. 2000; Goodell 2020; Fukasawa 2021). White rot is mainly associated with deciduous trees and brown rot with conifers, while showing signs for generalism (Schwarze et al. 2000; Krah et al. 2018). In the primary and intermediate stages, brown and white rot fungi dominate the decomposition, whereas soft rots find more suitable conditions towards late stages (Fukasawa 2021). In the case of timber that is used or stored outdoors, brown, white, or soft rot fungi might prevail during degradation depending on the conditions (Schmidt 2010). Even more dangerous is the fungal degradation of indoor wood. As currently mainly softwood is used for construction, brown rot fungi cause the biggest threat (Schmidt 2010; Kubicek 2012).

White rot fungi are a heterogeneous group of Basidiomycota, mostly from the *Agaricomycetes* (Savory 1954; Blanchette 1995). In white rot, the lignin is metabolized while some cellulose remains, causing the white appearance (Li et al. 2022). They can be further divided into selective and simultaneous delignifiers (either first degrading the lignin and then the cellulose or both in parallel) (Schwarze et al. 2000).

**Fig. 2:**
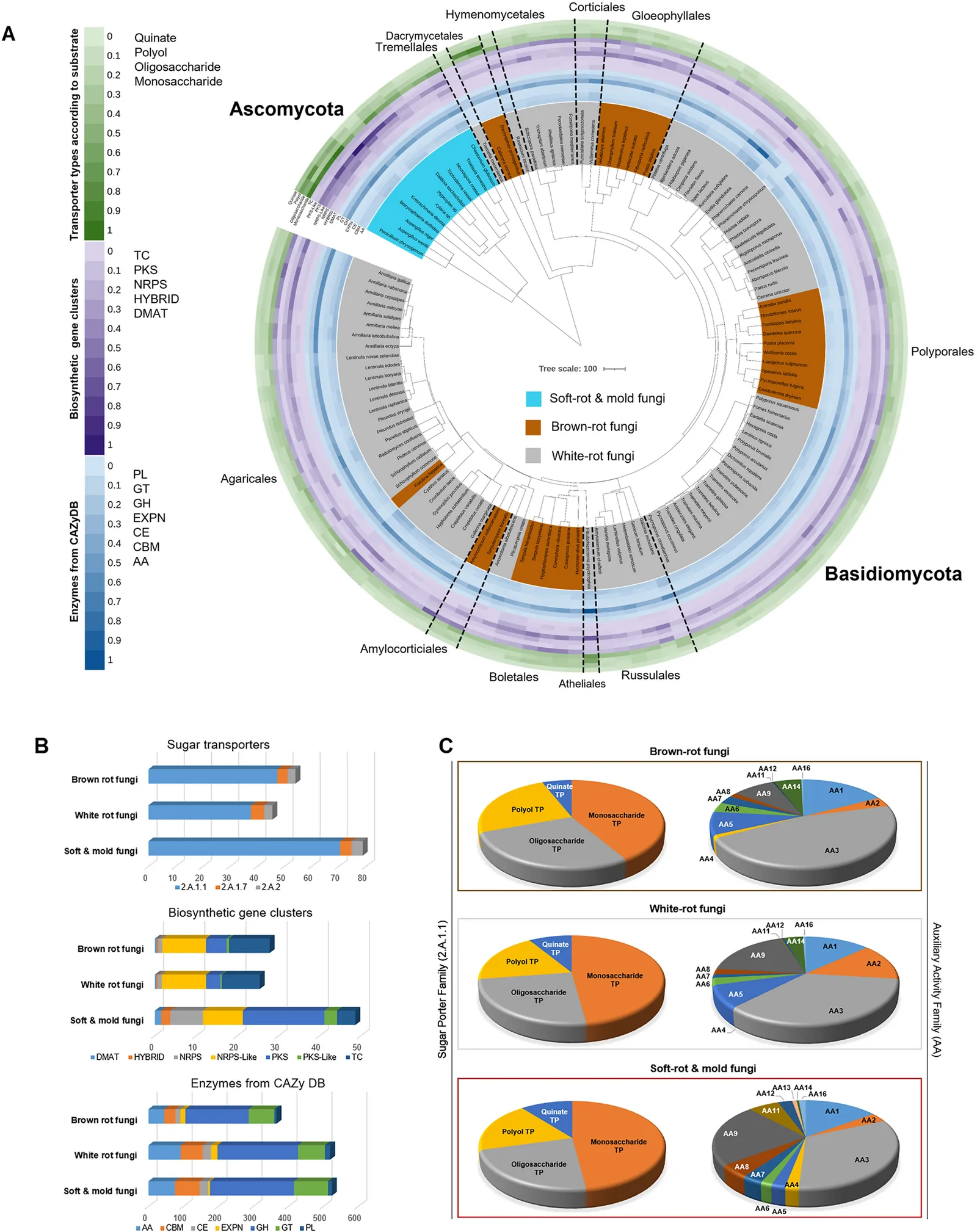

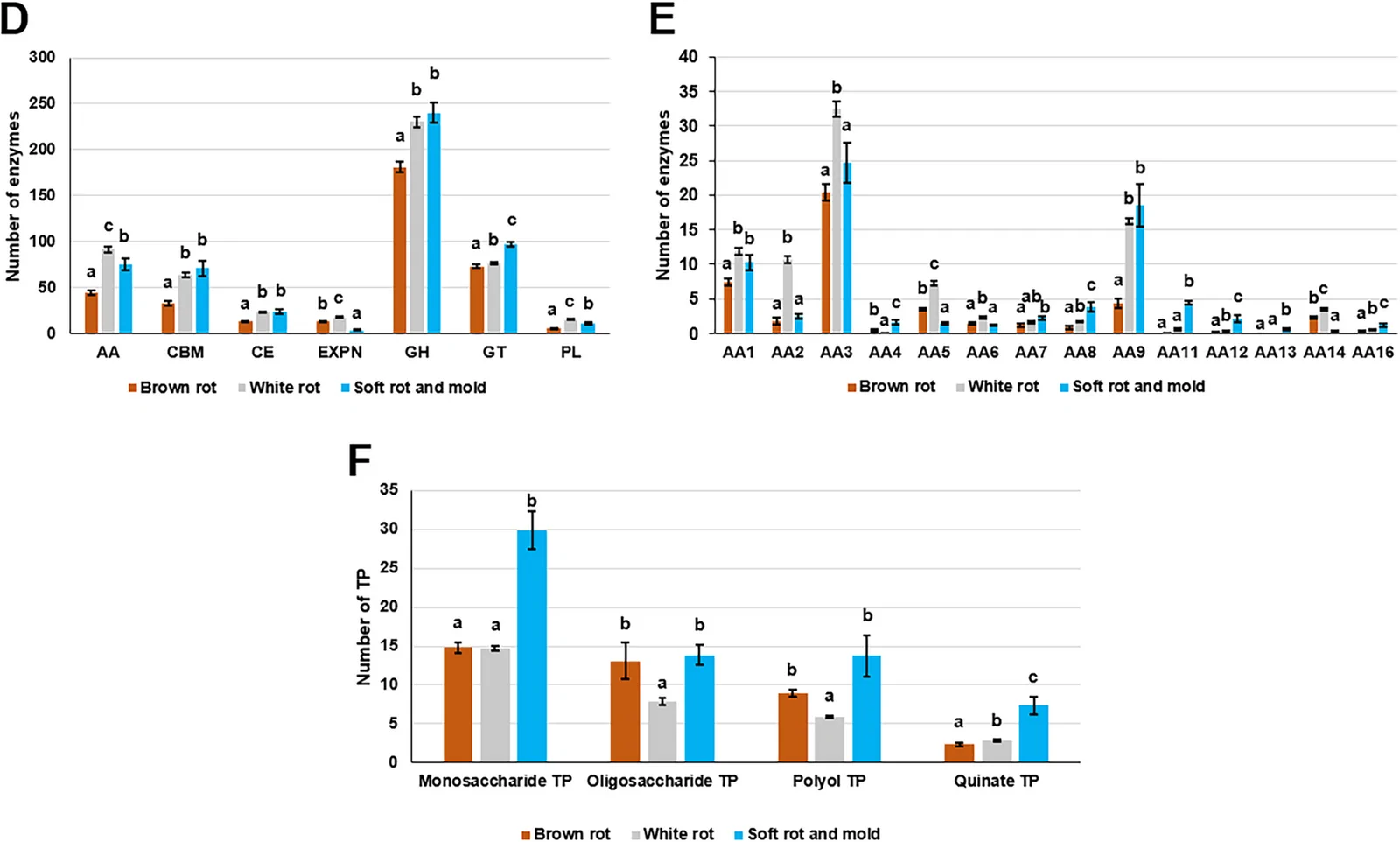
Overview of phylogenetic relationships, sugar transporters, biosynthetic gene clusters, and enzymes of brown rot, white rot, soft rot, and mold fungi. **A** Phylogenetic tree of selected rot species (including a few typical “molds”) created with TIMETREE5 (https://timetree.org/). Color shades indicate the predicted abundancy of transporter types according to substrate (green), biosynthetic gene clusters (violet), and enzymes from CAZy DB (dark blue). **B** Average number of genes per species encoding sugar transporters (top), biosynthetic gene clusters (middle), and CAZymes (bottom) of the three fungal groups. **C** Breakdown of genes encoding sugar transporters and enzymes from (**B**). Average share of sugar transporters from family 2.A.1.1 according to the substrate: monosaccharide transporters, oligosaccharide transporters, polyol transporters, and quinate transporters from the sugar porter family (left) and different Auxiliary Activity families (right) of the species belonging to the three fungal groups. **D** Average number of genes per species encoding enzymes belonging to different families from CAZy DB. **E** Average number of genes per species encoding enzymes from different Auxiliary Activity families. **F** Average number of genes per species encoding sugar transporters according to substrate. The data shown here should be taken with caution, as they are based on current (and often incomplete) genome annotations, and there is some “gray” spectrum of rot types (Schilling et al. 2020). Moreover, given that brown rot fungi may have evolved repeatedly from white rot ancestors and soft rot fungi are all Ascomycota while the former two groups are Basidiomycota, the observed differences among groups may partly reflect the evolutionary histories. 2.A.1.1, Sugar Porter family; 2.A.1.7, Fucose:H + symporter family; 2.A.2, Glycoside-Pentoside-Hexuronide:Cation symporter family. AA, Auxiliary Activity family; CBM, Carbohydrate-Binding Module family; CE, Carbohydrate Esterase family; DMAT, dimethylallyltryptophan synthase; EXPN, Expansins; GH, Glycoside Hydrolase family; GT, Glycosyl Transferase family; HYBRID, PKS & NRPS; NRPS, nonribosomal peptide synthetase; PKS, polyketide synthase; PL, Polysaccharide Lyase family; TC, terpene cyclase; TP, transporter. Information on sugar transporters, biosynthetic gene clusters, and CAZymes was retrieved from the JGI MycoCosm (https://mycocosm.jgi.doe.gov/mycocosm/home) database. Data are means ± standard error. Statistical differences were assessed using ANOVA followed by a Tukey *post hoc* test. Significant differences with *p* < 0.05 are shown with different letters

Brown rot fungi are also Basidiomycota (Blanchette 1995; Schwarze et al. 2000; Arantes et al. 2012), the majority of them belonging to the Polyporaceae (Schwarze et al. 2000). In brown rot, cellulose and hemicellulose are degraded without substantial removal of lignin (Floudas 2021; Fukasawa 2021), which therefore accumulates within the middle lamellae of the plant cell walls and gives the residual wood a brown and cuboid texture (Fukasawa 2021). This degradation can be attributed to untargeted but fast disintegration by hydroxyl radicals produced during the Fenton reaction, leading to rapid strength loss (Hyde and Wood 1997; Arantes et al. 2012). Brown rot as a fungal life style is believed to have evolved multiple times from a white rot common ancestor and is associated with a convergent loss of lignocellulose-degrading enzymes, but studies indicate that their radical-based strategy, supported by secondary metabolites (SMs), allows them to modify the lignin enough to efficiently access and degrade the carbohydrates without substantial removal of the lignin (Floudas et al. 2012; Kohler et al. 2015; Floudas 2021) (Fig. 2). Based on field studies of wood-rotting fungi in North America, it was estimated that around 7% of the wood-rotting fungi cause brown rot (Gilbertson 1980). However, due to the prevalence of Gymnosperms, they dominate decay in the global north.

“Soft rot” can be caused by a variety of Ascomycota as well as some Basidiomycota and is characterized by the preferred growth of the hyphae within the secondary plant cell wall, which manifests by typical cavities oriented longitudinally to the cell axis (Schwarze et al. 2000). Soft rot often occurs in wet or submerged wood, usually being restricted to the surface and emerging as a soft, decolorized surface layer (Savory 1954). Comparable to brown rot, cellulose and hemicellulose are preferentially degraded (Eaton and Hale 1993; Fukasawa 2021). Nevertheless, some soft rots degrade ~ 20% of the lignin (Nilsson et al. 1989).

A clear distinction between the three classic rot types is often not possible (Riley et al. 2014), and in addition to the presence/absence of certain genes and morphological appearance, other criteria such as chemical indicators like lignin loss relative to density loss and the change in wood dilute alkali solubility might also be considered (Schilling et al. 2020). The existence of this “gray” spectrum of rot types suggests that differences among decay groups should be interpreted taking into account their evolutionary histories, particularly because brown rot fungi have evolved repeatedly from white rot ancestors, and soft rot fungi are Ascomycota while the other two groups are Basidiomycota.

## Molecular mechanisms of wood degradation

Wood mainly consists of polysaccharides (predominantly cellulose and hemicelluloses) and the polyphenolic lignin (Zhu and Li 2024) (Fig. 1). The enzymes responsible for polysaccharide and lignin degradation are classified according to their reaction mechanism and structure (and not regarding the substrate they degrade) in the CAZy database (DB) (http://www.cazy.org/; (Drula et al. 2022)). Not long ago, an automated CAZyme and substrate annotation tool has been developed for substrate prediction (Zheng et al. 2023). In order to evaluate how the strategies of lignin and polysaccharide degradation of the three fungal rot types are reflected in their enzymatic machinery on genome level, we compared the main families and different types of enzymes by compiling CAZyme information from the annotated genomes available in the JGI (Joint Genome Institute) MycoCosm DB (https://mycocosm.jgi.doe.gov/mycocosm/home) (Fig. 2). The selected species of brown rot and white rot fungi form a polyphyletic group in the phylum Basidiomycota (Fig. 2A; Binder and Hibbett 2006; Pildain et al. 2018), with brown rot fungi belonging to the classes *Agaricomycetes* and *Dacrymycetes*, and the white rot fungi to the classes *Agaricomycetes* and *Tremellomycetes*. It is important to note that the intention of the present genome survey is to provide an exploratory comparison among fungal decay strategies, rather than a formal phylogenetically controlled comparative analysis, which represents an important direction of future research. Regarding the absolute number of wood-degrading enzymes, white rot and soft rot fungi have significantly more CAZyme genes than brown rot fungi (Fig. 2B), as observed previously (Floudas et al. 2012; Kameshwar and Qin 2018a). Moreover, white rot and soft rot fungi have on average more genes from the CBM (carbohydrate-binding modules) families than brown rots (Fig. 2D) (Kameshwar and Qin 2018a). CBMs are not catalytically active, but facilitate the binding of enzymes to their substrates. A meta-analysis of available genes in the fungal genomes according to rot type tries to display the underlying strategies of the different rot types to access the cellulose, which is restricted by coatings of hemicelluloses and lignin:

The degradation of cellulose—a linear polysaccharide of β-(1 → 4) linked d-glucose units—involves a synergistic action of cellulases: endoglucanases cleave internal β-(1 → 4) glycosidic bonds, exoglucanases release cellobiose from chain ends, and β-glucosidases hydrolyze cellobiose into glucose (Contreras et al. 2020). These subtypes of cellulases are classified into several glycoside hydrolase (GH), CBM, and auxiliary activity (AA) families of enzymes included in the CAZy DB (Rytioja et al. 2014; Sengupta et al. 2023) and exhibit distinct catalytic mechanisms, enabling efficient cellulose breakdown (Sidar et al. 2020). GH-type cellulases are more effective on amorphous cellulose, while the AA-family lytic polysaccharide monooxygenases (LPMOs) are more specialized for the crystalline regions (Uchiyama et al. 2022).

Hemicelluloses are non-crystalline polysaccharides typically with a β-(1 → 4) linked backbone made up of diverse sugars that are often branched and carrying substitutions (Agger et al. 2014). Their degradation requires a correspondingly diverse array of enzymes from GH, carbohydrate esterase (CE), CBM, and AA families working together to either cleave the hemicellulose backbone (including endo-β−1,4-xylanases, β-xylosidases, and endo-mannanases, belonging to GH; (Rytioja et al. 2014)) or remove side-chain decorations (like α-l-arabinofuranosidases and acetylxylan esterases, belonging to GH and CE) (Kameshwar and Qin 2018b; Shrivastava et al. 2023). The specificity and synergistic action of these hemicellulases facilitate efficient polymer breakdown. As for the cellulases, white rot and soft rot fungi possess a larger set of genes encoding putative hemicellulose- (and pectin-) active enzymes from several GH and CE families (Rytioja et al. 2014) (Fig. 2B).

Lignin is a heteropolymer made up of aryl-C3 units linked together via ether C-O and C-C bonds (Bugg 2024). It is formed in plants by radical polymerization of monolignol precursors (ρ-coumaryl alcohol, coniferyl alcohol, sinapyl alcohol), which can link together in different ways (Ralph et al. 2004). The fungal solution to this problem of highly recalcitrant substrate bonds and randomized surfaces was equally “radical”: Four main classes of fungal lignin-degrading enzymes have been developed (Martínez et al. 2009; Moiseenko et al. 2021): (1) lignin peroxidase, (2) manganese peroxidase, (3) versatile peroxidase, and (4) laccase. Laccases belong to the multi-copper oxidases and the other three to the heme-containing class II peroxidases (PODs). Both groups in turn belong to the oxidase super-family (Cagide and Castro-Sowinski 2020). Laccases could be further separated into laccases *sensu stricto *and laccase-like proteins, both assigned to the subfamily AA1 in the CAZy DB (Floudas 2021). The class II PODs are classified into the subfamily AA2, and their evolution towards higher redox potential from generic peroxidase (GP) over manganese peroxidase (MnP) to versatile peroxidase (VP) and lignin peroxidase (LiP) was suggested (Floudas 2021). Some authors consider further enzymes as ligninolytic, such as the dye-decolorizing peroxidases originally described in the ascomycete *Geotrichum candidum* (Kim and Shoda 1999) and later also in the white rot fungus *Pleurotus ostreatus* (Fernández-Fueyo et al. 2014), but their role remains uncertain (Floudas 2021). The initial step of fungal biodegradation of lignin yields different aromatic structures, including monolignols (Pollegioni et al. 2015; Becker and Wittmann 2019; Cagide and Castro-Sowinski 2020); for further details regarding the chemistry behind lignin bond cleavage, see Bugg (2024). The subsequent metabolic processes until the aromatic structures are fully mineralized to CO_2_ and H_2_O are still unclear in many aspects but might include decarboxylation, aromatic ring cleavage, and—after further modifications—entry into the central metabolism likely via Succinyl-CoA or Acetyl-CoA (Andlar et al. 2018; del Cerro et al. 2021). The so far analyzed genomes of brown rot fungi are mostly devoid of genes coding for PODs and often reduced in genes coding for laccases compared to white rot fungi (Floudas et al. 2015). While there is extensive experimental evidence that laccases (AA1) and PODs (AA2) are directly involved in lignin degradation, enzymes belonging to other AA classes have indirect contributions, such as H_2_O_2_ production (oxidases and dehydrogenases of families AA3 and AA5) and quinone reduction (AA6) (Floudas 2021). The latter are often summarized by the term lignin-degrading auxiliary enzymes (Hatti-kaul and Ibrahim 2013; Janusz et al. 2017).

Our meta-analysis (Fig. 2C) supports that white rot genomes contain a substantially higher average number of enzymes belonging to the ligninolytic families AA3 and AA5 and a higher proportion of enzymes from the families AA2 (PODs) than the other two wood-degrading fungal groups, consistent with their ability to degrade lignin (Fig. 2E). From an evolutionary point of view, this fact supports the idea that brown rot and soft rot fungi, which mainly metabolize the non-lignin components of wood (cellulose and hemicelluloses), could have reduced these families during the development of their respective strategies for wood-decay following the establishment of seed plants during the Late Paleozoic (Cai et al. 2024). Moreover, white and brown rot fungi can also be distinguished by the number of enzymes belonging to the AA8 (e.g., cytochrome domains of cellobiose dehydrogenases (CDHs) supporting LPMOs; (Eastwood et al. 2011)) and AA9 (cellulolytic LPMOs) families, which are expanded in the case of the white rot fungi (Levasseur et al. 2013; Rytioja et al. 2014), although both families are actually proportionally most abundant in soft rot fungi (Fig. 2C). On the other hand, Ascomycota can only degrade the softer section of wood, with a low proportion of lignin. One reason for this is likely the lower T1 redox potential in their laccases compared with white and brown rot fungi (Rodgers et al. 2010; Kameshwar and Qin 2018b). Therefore, they have wood rot activities, but at a much lower rate than basidiomycete wood-degrading fungi (Asemoloye et al. 2021). Aligned with this notion, lignin modification rather than mineralization was suggested for soft rot fungi (Weng et al. 2021).

The enzymatic and oxidative opening of the wood cell wall ultimately serves one central purpose: the release of soluble carbon sources that can be imported and metabolized by the fungus.

## Sugar uptake and nutrient acquisition

Once lignocellulose has been depolymerized outside the hypha, the resulting soluble sugars must be taken up across the fungal plasma membrane before they can fuel growth, maintenance, and further decay. The uptake of mobilized sugars across the plasma membrane by transporter proteins is therefore a major step in fungal conversion of plant biomass. Based on phylogenetic analysis, most of the sugar transporters from filamentous fungi (99%) belong to the major facilitator superfamily (MFS, TC 2.A.1) as categorized in the Transporter Classification Database (TCDB) (Nogueira et al. 2020; Saier et al. 2021). These are typically twelve-transmembrane-domain proteins with specificity for structurally and chemically related mono- or oligosaccharides. However, of the many transporters present in the fungal genomes, in most cases, the substrates for only a fraction are known. In the soft rot ascomycetes *Aspergillus nidulans*, *Aspergillus niger*, and *Trichoderma reesei*, for example, only 14 out of 112, 11 out of 114, and 16 out of 64 putative sugar transporters, respectively, have been characterized so far (Nogueira et al. 2020; Xu et al. 2024). Although several MFS sugar transporters have been found in the genome of white rot fungi (Suzuki et al. 2012; Xie et al. 2021), there is only one report of functional characterization in these organisms: *PsHxt1*, a monosaccharide transporter from *Phanerochaete sordida*. Its overexpression led to the discovery of its ability to transport a variety of monosaccharides: glucose, fructose, mannose, xylose, and galactose (Mori et al. 2019).

The available data from the JGI MycoCosm indicate that soft rot fungi, on average, have more transporters belonging to the sugar porter family (TC 2.A.1.1) and the glycoside-pentoside-hexuronide:cation symporter family (TC 2.A.2) than brown or white rot fungi (Fig. 2B). Furthermore, soft rots, on average, have approximately twice as many monosaccharide and quinate transporters than the other two groups, but fewer oligosaccharide and polyol transporters (Fig. 2F). The fact that brown rot and white rot fungi have a higher proportion of oligosaccharide transporters could be interpreted as a way to bypass carbon catabolite repression (CCR), but also to become more competitive against co-inhabiting microorganisms with less elaborate enzyme systems relying on monosaccharides. The lower disaccharide assimilation capacity of Ascomycota (to which soft rot fungi belong) has already been noted in the past, having typically less extensive repertoires of CAZymes (Floudas et al. 2012).

While the collectivity of all sugar transporters present in a fungus likely is an excellent readout for its niching behavior and describes its substrate preferences, our understanding of these gatekeepers is far from what would be necessary to do so. Many more transporters will need to be characterized and structures obtained to enable robust predictions of substrate specificities from genomic information. However, first programs have been published in this direction (Kroll et al. 2024) that allow the selection of putative substrates from candidate sugar transporters, making their experimental characterization more efficient and manageable.

Which transporters are expressed at a given stage of decay, however, depends on nutrient sensing, carbon availability, and the regulatory networks that coordinate extracellular enzyme production with intracellular uptake capacity.

## Regulation of plant cell wall degradation

The deployment of plant cell wall-degrading enzymes and sugar transporters is tightly coordinated with nutrient availability. Fungi have therefore evolved transcriptional networks that selectively express the required genes according to the current substrate and carbon status. While extensive information is available on characterized transcription factors in the phylum Ascomycota (Amore et al. 2013; Kerkaert and Huberman 2023), in-depth studies for true wood-degrading Basidiomycota are scarce. A primary and highly conserved mechanism is CCR, which inhibits the secretion of CAZymes when simple sugars are present, in order to save metabolic energy. The transcription factor CreA/CRE-1 is the main regulator of CCR. It has been characterized in several Ascomycota and is conserved in Basidiomycota (Todd et al. 2014; Daly et al. 2019), in whose genomes copies of the CreA-related SYGGRT binding site have been found (Rytioja et al. 2014). Moreover, deletion of CRE1 in the white rot *Pleurotus ostreatus* was found to lead to enhanced cellulose degradation (Yoav et al. 2018). On the other hand, Todd et al. (2014) did not find basidiomycete orthologs for several well-known ascomycete transcriptional activators with a role in the utilization of plant biomass, such as ARA-1, PDR-1/2, and XLR-1 (Todd et al. 2014). This fact suggests that most basidiomycete regulators have developed after the split from Ascomycota, and their molecular mechanisms might differ significantly. However, the activator of cellulase expression 3 (ACE3), described in *Trichoderma reesei* as a regulator of lignocellulose degradation (Häkkinen et al. 2014), has also been found in Basidiomycota (Benocci et al. 2017). Marian et al. (2022) recently identified the Zn_2_Cys_6_-type transcription factor Roc1 as key regulator of cellulose degradation in the white rot fungus *Schizophyllum commune*. This transcription factor is highly conserved in the phylum Basidiomycota, but was only found in members of the class *Agaricomycetes*. Expression analysis on lignocellulose indicated that Roc1 orthologs from *Agaricus bisporus* and *Dichomitus squalens* might have a conserved role (Marian et al. 2022). More recently, the ortholog of *S. commune* Roc1 in *D. squalens*, named *Ds*Ace3, was characterized and found to be essential for cellulose degradation (Ramos et al. 2026). Another transcription factor involved in nutrient regulation that was recently characterized in a basidiomycete is GCN4 from *Ganoderma lucidum*. It plays an important role in nitrogen utilization and fungal growth by maintaining the amino acid content under nitrogen-limiting conditions (Lian et al. 2023). However, although the repertoire of potential transcription factors of wood-degrading Basidiomycota has been proposed by in silico studies (Shelest 2017; Okal et al. 2020; Todd et al. 2022), where basidiomycetes have, on average, fewer transcription factors than ascomycetes (283 versus 366; (Todd et al. 2014)), much more research is needed on the regulatory mechanisms involved in plant biomass degradation in these fungi.

## Secondary metabolites in wood decay, communication, and competition

**Fig. 3:**
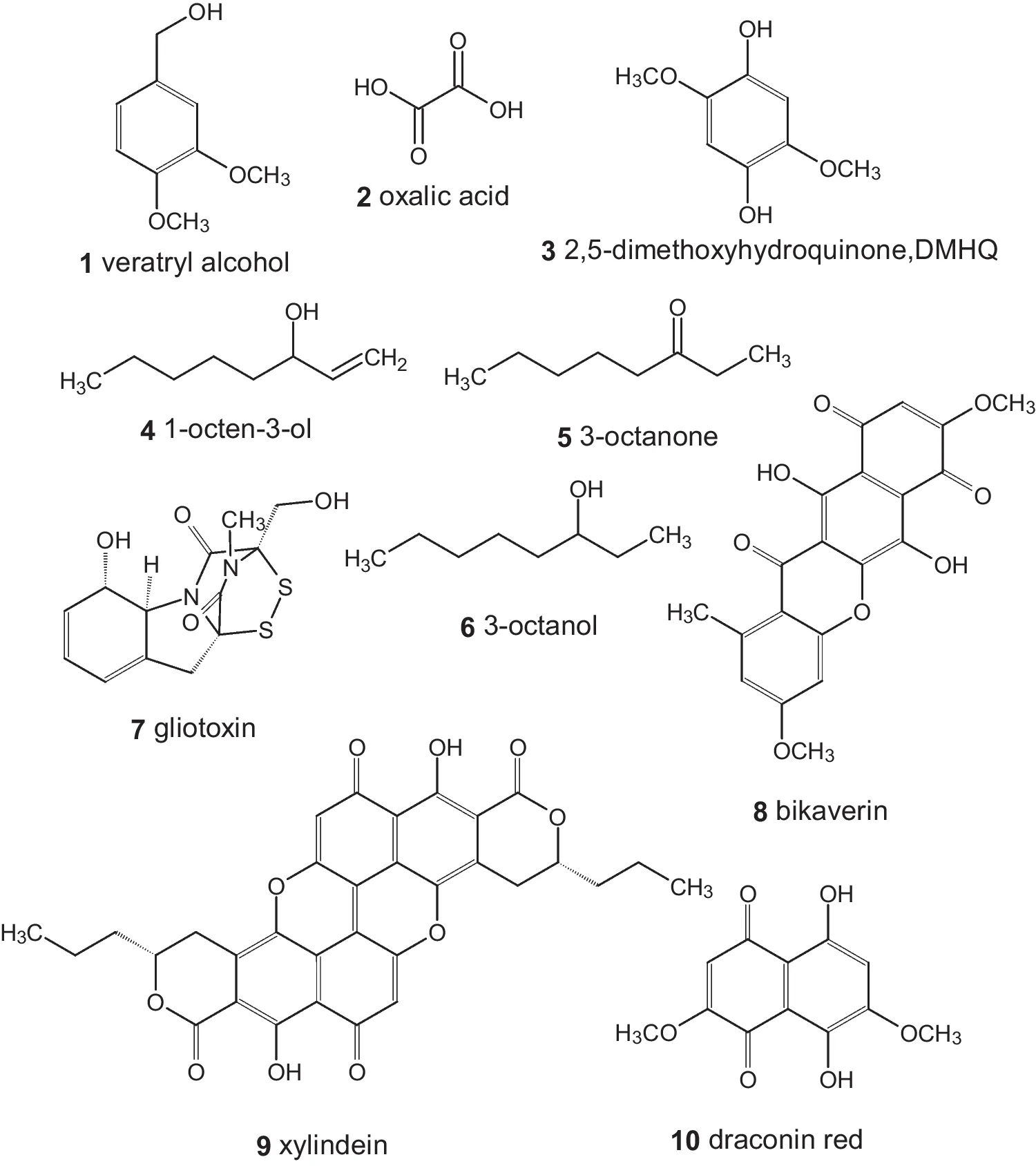
Secondary metabolites produced by wood-decaying fungi and involved in wood degradation and discoloration. Drawn with ChemSketch

Beyond extracellular enzymes, sugar transporters, and their regulatory networks, SMs represent an additional chemical layer in fungal wood decay. They can modulate redox reactions, metal availability, oxidative stress, and interactions with surrounding microorganisms, thereby supporting degradation as well as competition and communication. Wood-degrading fungi utilize such compounds to support, and in some cases partially replace, enzymatic breakdown of wood structural components. These metabolites include organic acids, quinones, and phenolic compounds (Castaño et al. 2022). White rot fungi employ oxidative processes to oxidize and cleave lignin into smaller units. Key SMs involved in this process include veratryl alcohol (**1**, Fig. 3) and oxalate / oxalic acid (**2**, Fig. 3) (Ten Have and Teunissen 2001). Veratryl alcohol, a phenolic compound, acts as a mediator, stabilizing reactive intermediates and extending the range of lignin oxidation (Díaz-González et al. 2011). Oxalate, an organic acid, chelates metal ions, preventing inhibition of oxidative reactions by metal-catalyzed scavenging of reactive oxygen species (Gadd et al. 2014). Metabolomics studies in *Trametes versicolor* showed early sugar release, rapid phenolic catabolism, and late stage veratryl alcohol accumulation (Castaño et al. 2022).

In brown rot fungi, oxidative depolymerization is driven largely by Fenton chemistry, initiated when the SM 2,5-dimethoxyhydroquinone (DMHQ) (**3**, Fig. 3) reduces Fe^3+^ to Fe^2+^ (Arantes et al. 2012). This oxidative degradation is particularly effective at attacking the amorphous regions of cellulose, enabling rapid wood decomposition and also involves strong oxalate gradients established around the hyphae (Arantes and Goodell 2014). Beyond DMHQ, in‑planta metabolomics showed that brown rot fungi synthesize a diverse portfolio of SMs, including pyranones, furanones, carboxylic acids, and sugar-derived compounds (e.g., d‑arabitol, d‑xylitol, glyceric acid), which function in redox buffering, antioxidant defense, and antimicrobial activity during reactive oxygen species (ROS)-intensive decay (Riley et al. 2014; Castaño et al. 2021, 2022).

Biosynthetic gene clusters (BGCs) provide a genome-level indication of the potential chemical repertoire of wood-degrading fungi. BGCs are commonly classified according to their core biosynthetic genes, including polyketide synthases (PKS), non-ribosomal peptide synthetases (NRPS), hybrid PKS-NRPS clusters, dimethylallyltryptophan synthases (DMAT), and terpene cyclases (TC) (Khaldi et al. 2010; Robey et al. 2021). However, the presence of a predicted BGC does not necessarily mean that the corresponding pathway is expressed under wood-decay conditions or that the final metabolite and its ecological function are known. Therefore, the BGC data shown in Fig. 2B should be interpreted as biosynthetic potential rather than direct metabolite production. The current figure also defines the BGC panel as part of the genome-level comparison across brown rot, white rot, and soft rot / mold fungi.

The available BGC annotation from the JGI MycoCosm indicates that soft rot and mold fungi have the highest predicted secondary-metabolite potential, with approximately 49 BGCs per species on average, compared with approximately 28 in brown rot and 25 in white rot fungi. This difference is mainly driven by the high average number of PKS clusters in soft rot and mold fungi, about 20 per species, together with higher numbers of NRPS and hybrid PKS-NRPS clusters. In contrast, brown and white rot Basidiomycota retain relatively high numbers of NRPS-like and TC clusters, approximately 9–11 per species, respectively. This pattern suggests that soft rot and mold fungi may have a broader PKS- and NRPS-dominated chemical repertoire, reflecting their strong ecological interaction, stress adaptation, and competition capacity. Brown and white rot fungi, in contrast, may rely on a smaller but more specialized set of metabolites, including redox-active compounds and terpenoid-related chemistry, which are more directly connected to decay-specific redox reactions, substrate colonization, and defense against competitors.

Soft rot and mold fungi furthermore generate a diverse array of microbial volatile organic compounds (mVOCs) and polyketide-derived SMs, including the “C_8_ complex” (comprised of 1-octen-3-ol (**4**, Fig. 3), 3-octanone (**5**, Fig. 3), and 3-octanol (**6**, Fig. 3)), as well as various terpenes, alkenes, and methyl/ethyl ketones (Wilkins et al. 2000; Wadhwa et al. 2024). These metabolic signatures are highly characteristic, appearing in patterns that are strictly dependent on both the fungal species and the specific substrate on which they grow (Wilkins et al. 2000). While these SMs contribute little to the primary enzymatic deconstruction of lignocellulose, they are critical for environmental interaction and ecological success (Elhamouly et al. 2022; Wadhwa et al. 2024). Compounds including peptaibols, gliotoxin (**7**, Fig. 3), and bikaverin (**8**, Fig. 3), alongside various siderophores, function as antagonistic agents or signaling molecules that are frequently triggered during co-culture or physical contact (Rangel et al. 2021). Many of the underlying biosynthetic processes are governed by the master regulator velvet protein complex (VelB-VeA-LaeA), a global regulatory system that synchronizes metabolite production with environmental stress and fungal development (Bayram and Braus 2012). Overall, these compounds function as a sophisticated “chemical language” used for long-distance signaling, mediating competitive interference, and the spatial structuring of fungal communities (Krause et al. 2020; Rangel et al. 2021). By altering the surrounding chemical landscape, these metabolites enable fungi to navigate and dominate microbially crowded environments, particularly in habitats with variable moisture levels.

Compared with Ascomycota, where PKS- and NRPS-derived SMs have been studied extensively, secondary metabolism in wood-degrading Basidiomycota remains less systematically connected to BGCs, pathway regulation, and ecological function. However, Basidiomycota are known to produce diverse SMs, especially terpenoids and meroterpenoids, but also polyketides, alkaloids, phenolics, and other nitrogen-containing compounds (Stadler and Hoffmeister 2015; Sum et al. 2023). Thus, the current knowledge gap is not whether Basidiomycota produce SMs, but rather which predicted pathways are active during wood colonization and how their products contribute to degradation, communication, or competition.

Within basidiomycetes, wood-rotters have also evolved strategies for antimicrobial warfare and nutrient sequestration. White rot basidiomycetes deploy a suite of bioactive terpenoids, phenolics, and β-glucans that offer antioxidant and antimicrobial protection (Pinar and Rodríguez-Couto 2024). In contrast, brown rot fungi seem to prioritize PKS-derived pyranones and furanones to shield excess sugars and stabilize the environment during oxidative decay (Castaño et al. 2022). Recent natural-product studies from Basidiomycota-focused groups further highlight that mushroom-forming fungi remain chemically underexplored (Gressler et al. 2021; Han et al. 2023; Schmidt et al. 2024); however, for many newly described compounds, their direct role in wood degradation or fungal competition is still unclear. Current advancements in omics and synthetic biology are now allowing to activate previously “silent” pathways, directly connecting BGC potential to specific ecological roles across different wood-decaying guilds (Flak et al. 2020; Rendsvig et al. 2020).

## Threats to living trees and wood products

**Fig. 4:**
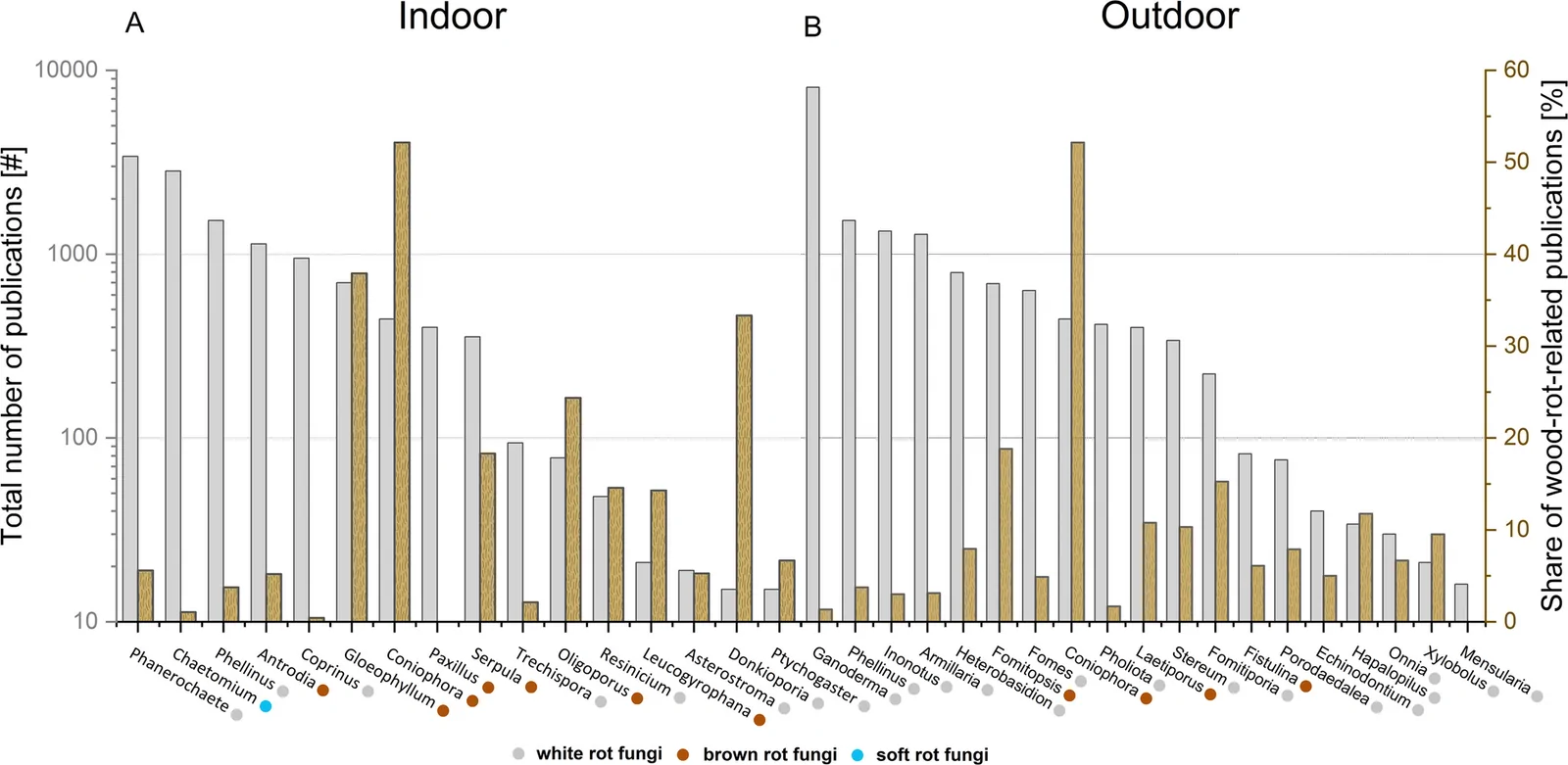
Total and wood rot-related publications of fungal genera posing a threat to wood in indoor environments according to Schmidt (2007) (**A**) and living trees according to Gonthier and Nicolotti (2013) (**B**). The genera are marked as white, brown, or soft rots. The total number of publications regarding certain fungal genera was obtained by the searching query [article title OR abstract OR keywords = genus name] and the subset of publications regarding the respective genus addressing wood rot by [[article title OR abstract OR keywords = genus name] AND [article title = “wood”] AND [all fields = “rot”] AND [year ≥ 2000]]. The resulting wood-rot-related publications were spot-checked to validate the queries, but a complete assignment of all publications on wood rot cannot be guaranteed. Furthermore, it should be noted that the same genus names as in Schmidt (2007) and Gonthier and Nicolotti (2013) have been used, so that publications using synonyms as well as older or more recent names were excluded. All queries were made in the Scopus database (www.scopus.com) on 23.02.2025

To provide an overview of the state of research on threats for standing, lying, or used wood, we performed a meta-analysis of the literature for fungal genera frequently found on wood in indoor and outdoor environments (acc. to Schmidt 2007; Gonthier and Nicolotti 2013) (Fig. 4). While construction wood can be severely damaged by saprobes, the biggest threat for living trees are parasites. The death of living trees can, however, even bring advantages for other plants competing for light, space, water, and nutrients and make the dead wood available as substrate for other microbes or habitat for animals (Marcot 2017). Overall, the economic damage caused by wood-degrading fungi in indoor wood is higher than in living trees (Worrall), which might explain the higher wood rot-related share of publications (Fig. 4A vs. B). However, already Li et al. (2022) noted that most articles about wood-degrading fungi appear in the research disciplines of biotechnology / applied microbiology, environmental sciences, and microbiology, whereas forestry only ranks 5th (Li et al. 2022).

The genus *Coniophora* shows the highest share of wood rot-related publications. It is causing brown rot of living trees and wood products and *C. puteana* is widely used as a model organism in the EN 113 standard (EN 113-2 2021), specifying laboratory procedures for evaluating wood resistance to Basidiomycota, which is likely the cause for the many citations. Although species of *Coniophora* are most commonly listed as saprotrophs, some sources describe them as cause of tree death (Whitney and Myren 1978; Lännenpää et al. 2008). Likewise, *Gloeophyllum trabeum* is also used as a representative brown rot species within the EN 113 (EN 113-2 2021), which explains the comparatively high number of wood rot-related studies associated with this genus. The genus *Donkioporia* accounts for the third-highest share of wood rot-related publications, primarily due to its species *D. expansa*, commonly known as the “oak polypore,” which in recent decades has been recognized as an economically important indoor fungus (Kleist and Seehann 1999; Schmidt 2007). The main focus regarding outdoor wood rot is *Fomitopsis*. The brown rot *F. pinicola* is highly abundant on conifers in the temperate and boreal Northern Hemisphere and leads to substantial mass loss of heart and sapwood (Holsten et al. 2001; Son et al. 2011; Gonthier and Nicolotti 2013).

The genus *Ganoderma* attracts the most overall research interest of the considered wood-degrading fungi (Fig. 4), albeit with rare wood-related focus. *Ganoderma* species have an enormous biotechnological relevance, which was made use of early on in their history as cultivated medicinal mushrooms in Asia (Seethapathy et al. 2023). Still today the industrial interest is substantial, especially for cosmetics, health care, and food products (Hapuarachchi et al. 2018; Seethapathy et al. 2023), but more recently also in the area of myco-materials (Huang et al. 2024). *Phanerochaete* and *Chaetomium* follow with the second and third-highest counts of publications, respectively. The prominence of the former mainly reflects the exceptional research interest in *Phanerochaete chrysosporium*, a model white rot species used to study, for example, lignin biodegradation (Jatwani et al. 2025). In addition, its genome sequence was among the first available for any basidiomycete, significantly advancing our understanding of fungal lignocellulose metabolism (Kersten and Cullen 2007). The soft rot-causing genus *Chaetomium*, with a smaller share of wood rot-related studies, belongs to the Ascomycota. Comprising over 400 species, it is taxonomically complex (Læssøe and Petersen 2019). Research interest mainly stems from its role as a biological control agent against plant pathogens (Lavanya et al. 2025) and its capacity to degrade cellulose and produce diverse bioactive metabolites (Wang et al. 2016). Despite having a relatively high number of total publications, *Paxillus* is not represented in studies on wood rot (Fig. 4A). However, *P. involutus*, one of the best-studied ectomycorrhizal fungi, is capable of degrading organic matter in plant litter through a brown rot-type mechanism (Rineau et al. 2012), which may explain why this genus is occasionally found in buildings, as reported by Huckfeldt and Schmidt (2006).

Depending on the environmental conditions and tree species, the parasitic fungal species that pose a threat to forests vary. Moreover, age also matters, as trees younger than 60 years can be affected by root and butt rots (*Armillaria*, *Ganoderma*, *Heterobasidion*, *Onnia*, and some *Phellinus* species in Fig. 4B), whereas heart rot only plays a minor role (Tainter and Baker 1996). Despite a substantial decrease in heart rot of timber—caused by the harvest of younger trees in the course of the twentieth century—Tainter rated heart rot “the most damaging of all types of tree diseases” for deciduous and coniferous trees. However, when it comes to economic damage, this view is challenged. Root and butt rots, specifically from the genus *Heterobasidion,* are referred to as the most destructive diseases of conifers in the northern hemisphere (Asiegbu et al. 2005; Arhipova et al. 2011; Gonthier et al. 2012). Furthermore, different *Armillaria* species are causing the death of various host trees globally in large numbers (Kim et al. 2022). Considering this, the identified number of wood rot-related publications appears very low for both of them. The same is true for the genus *Serpula*, given that *S. lacrymans* is a well-known “house rot” fungus and one of the most destructive and difficult-to-control indoor wood-degrading species (Schmidt 2007). Moreover, while *C. globosum* is a model fungus used in standardized tests assessing wood-preservative efficacy against soft rot micro-fungi (EN 807 2001) and is known to synthesize toxic compounds, our analysis suggests that the ecology and potential health relevance of other indoor *Chaetomium* species remain underexplored.

## Secondary metabolite-driven threats to wood quality and durability

In the context of wood degradation, SMs become a threat mainly when they reduce the value, durability, or usability of wood, rather than through direct toxicity to humans. Therefore, the most relevant risks are not primarily associated with the ingestion of toxic fruiting bodies, but with chemical changes induced in the wood itself, including discoloration, staining, acidification, metal chelation, and the promotion of oxidative decay reactions (Arantes et al. 2012; Gadd et al. 2014; Morris et al. 2021). These effects may be especially relevant in stored timber, construction wood, historical wooden objects, and wood products where visual quality, mechanical integrity, and long-term durability are important.

One visible consequence of fungal secondary metabolism is wood discoloration. Spalting is broadly defined as a fungal modification of wood color and pattern, including pigmentation, bleaching, and the formation of dark zone lines, which are mainly formed by melanized hyphae and often arise from antagonistic interactions between decay fungi. Specific pigments produced by wood-colonizing fungi illustrate this ambivalent effect: among the best-studied pigment-type spalting fungi are *Chlorociboria aeruginosa*, which produces the blue-green quinonic pigment xylindein (**9**, Fig. 3), *Scytalidium cuboideum*, which produces the red naphthoquinone pigment draconin red (**10**, Fig. 3), and *Scytalidium ganodermophthorum*, which produces a yellow pigment widely used in experimental fungal pigment studies but less completely characterized structurally (Weber et al. 2014). These pigments can penetrate or stain wood and have attracted interest as natural colorants for wood, textiles, and coatings. From a material perspective, fungal pigmentation is therefore context-dependent: in conventional timber and wood products, it is usually undesirable when a uniform appearance is required and can reduce commercial value, whereas controlled fungal pigmentation can be exploited for decorative spalting or natural colorant applications (Robinson et al. 2014; Morris et al. 2021). In addition to diffusible pigments, blue stain or sap stain fungi cause blue, gray, or black discoloration of sapwood mainly through dark melanized hyphae rather than through a single extracellular pigment; this type of staining often has limited effects on mechanical strength, but is considered a major aesthetic and economic defect because it lowers visual quality and market value (Humar et al. 2008; Uzunovic et al. 2008).

A second threat arises from small organic acids and redox-active metabolites secreted during decay. Oxalic acid is particularly important because it can acidify the local wood environment, chelate metal ions, and participate in iron- and manganese-dependent redox processes, as described above with respect to the chelator-mediated Fenton reactions.

These metabolite-driven processes can also contribute to continued or hidden deterioration after harvesting or installation, particularly when moisture conditions remain favorable. Pigments and melanized hyphae may signal prior or ongoing fungal colonization, while organic acids, quinones, and other low-molecular-weight redox compounds can facilitate the early chemical opening of the wood cell wall before extensive enzymatic degradation becomes visible (Arantes and Goodell 2014; Castaño et al. 2021). Therefore, SMs should be considered not only as products of fungal metabolism but also as early-acting chemical tools that can reduce wood quality, promote further colonization, and accelerate structural or aesthetic deterioration.

## Utilization of macro- and microscopic fungal traits

The loss of strength and stiffness of wood due to fungal degradation is a problem whenever the wood ought to fulfill load-bearing roles, such as in construction materials but also in native trees. For some applications, however, softening the wood is beneficial. This controlled fungal colonization is used to create so-called myco-wood for pencil and musical instrument manufacturing. Depolymerization of the lignin by white rot fungi increases the porosity of the wood, which is associated with enhanced sound absorption properties, better sharpenability (pencils), and impregnability of the resulting myco-wood (Stange and Wagenführ 2022).

Spalting, defined as a change in wood color caused by the colonization of brown, white, or soft rot fungi, is another utilization potential (Robinson et al. 2014; Morris et al. 2021). Humans have made use of this phenomenon for at least 550 years by utilizing spalted wood as an aesthetic element (Blanchette et al. 1992). Nowadays, efforts are being made to produce spalted wood under optimized conditions, which could increase its value by one order of magnitude compared to firewood (Morris et al. 2021).

In some sense, the disintegration of wood into wood particles can be inversed by letting a fungus colonize the latter. The mycelial network connects to all the wood particles, seeking to digest them, thereby creating new structural integrity (Girometta et al. 2019). If the fungus is deactivated before complete degradation of the substrate, a mycelium-bound composite (MBC) is formed. Apart from wood, various other organic particles or fibers can be used as compounds in these materials (Huang et al. 2024). Together with the adjustment of growth conditions and post-processing, the variety of different fungus-substrate combinations leads to versatile products for applications such as packaging, sound absorption, thermal insulation, or architectural design (Appels et al. 2019; Meyer et al. 2020; Aiduang et al. 2022a). The fungal species used for successfully fabricating stable composite materials are almost exclusively white rots (Huang et al. 2024). Selection criteria in favor of white rot fungi are their tough mycelia, fast colonization rates, and ability to degrade a variety of organic substrates (Manan et al. 2021; Aquino et al. 2022). The better mechanical and dimensional stability of MBCs from white- instead of brown rot was additionally highlighted (Sydor et al. 2022). However, whether the destabilization of the wood cell wall structures by the brown rot Fenton reaction (as discussed above) indeed affects the interconnection or structural stability of the wood particles too badly remains to be shown.

For the choice of suitable fungal strains for MBCs, growth rate, binding capacity, and tolerance to different growth conditions as well as non-toxicity should be considered (Sydor et al. 2022). The growth rate of fungi under defined conditions does not only vary between but also within species, making definite statements difficult (Dresch et al. 2015). However, some species are successfully used on different substrates in different laboratories (Aiduang et al. 2022a; Huang et al. 2024). A strongly branched, dense hyphal network (phalanx growth strategy) ensures good interconnection and helps inhibit contamination by other organisms (Sydor et al. 2022).

Finally, there are also applications where the product is not mycologically treated biomass or a composite of fungus and substrate, but the pure fungus itself. The most well-known and probably oldest example is the cultivation of edible and medicinal mushrooms, which are often wood decomposers (Stamets 2011). A more recent discovery is the utilization potential of pure mycelium, especially as a leather substitute (Vandelook et al. 2021; Whabi et al. 2024). Selection criteria for the fungal species involve the fast production of biomass on different substrates and mechanical characteristics determined by the composition and morphology of the hyphae (Vandelook et al. 2021; Whabi et al. 2024). Although many different species from different phyla were tested, the focus is often on well-known edible and medicinal mushrooms (Cartabia et al. 2021; Amobonye et al. 2023; Elsacker et al. 2023), giving them an advantage due to existing cultivation expertise.

When it comes to the binding capacity of fungal species, many authors attribute importance to di- or trimitic hyphal systems (Jones et al. 2020; Aiduang et al. 2022b; Sydor et al. 2022; Balaeș et al. 2023) (see also Fig. 1). While the enhanced mechanical properties of binding hyphae and especially skeletal hyphae (only present in di- and trimitic systems) are described, the abundance or even presence of them in MBCs has not been sufficiently studied so far (Bayer and McIntyre 2012; Aquino et al. 2022; Porter and Naleway 2022). Overall, the presence and markedness of relevant fungal traits should be evaluated for the desired application. For instance, a fungus with trimitic fruiting bodies does not guarantee three types of hyphae when put to use for mycelium material fabrication, and even when present, they might not play the same role as in fruiting bodies.

## Utilization of fungal enzymes, transporters, and metabolites

The potential of wood-degrading fungi to produce valuable enzymes and SMs can be used for many biotechnological applications in biofuel and bioethanol production, bio-based platform chemicals, pharmaceuticals and cosmetics, bioremediation, and improvement of byproduct yield (Asemoloye et al. 2021). Some of the enzymes produced by white rot fungi are involved in the synthesis of SMs like lactobionic acid, with great potential for application in cosmetics, food industry, and medicine (Ciullini et al. 2008; Nyanhongo et al. 2017; Piątek-Gołda et al. 2023). Furthermore, the lignin degradation properties of white rot fungi have uses in the biopulping process of the paper industry (Weng et al. 2021). In the area of environmental management, other applications of fungal lignin-degrading enzymes include wastewater and soil treatment (Rivera-Hoyos et al. 2013; Kües 2015; Arregui et al. 2019; Mäkelä et al. 2020), industrial waste management (Senthivelan et al. 2016), detoxification of industrial dyes and xenobiotic compounds (Rivera-Hoyos et al. 2013; Kües 2015), and treatment of environmental contaminants (Temporiti et al. 2022; Ghosh et al. 2023).

From an economic point of view, enzyme production is a bottleneck for the viability of biorefineries, especially for biofuel production (Klein-Marcuschamer et al. 2012). In this regard, “press water”, a byproduct of the mechanical drying of wood chips, commonly used for energy production, was shown to be a suitable medium supplement for several wood-degrading, enzyme-producing Basidiomycota (Reppke et al. 2022). This finding opened the possibility of combining biological wastewater treatment with other biotechnological applications. Another innovation for environmental management that deserves mention is known as “mycofiltration,” the use of fungal mycelium as a membrane to filter out contaminants, including microorganisms and pollutants (Stamets 2005; Mehta et al. 2017). Also here, the tough and highly interconnected mycelia of wood-degrading fungi have clear advantages.

In addition to lignin-degrading enzymes and CAZymes, membrane transporters are also very important for industrial applications (Soares-Silva et al. 2020). When sugar uptake is optimized by strain engineering, the utilization of hydrolysis products from renewable substrates such as lignocellulosic biomass into the cell can be increased or facilitated (Alonso et al. 2015). However, with only few exceptions (Mori et al. 2019), not much research has been done so far on the characterization of sugar transporters in wood-decomposing Basidiomycota, and thus, further studies are needed in this regard.

## Conclusions

In the present mini-review, we presented the fascinating group of wood-degrading fungi, being among the most influential biological drivers of carbon turnover in terrestrial ecosystems. Their success is based on highly diverse strategies for colonizing wood, acquiring nutrients, and interacting with competitors, reflected in distinct morphological traits, enzymatic repertoires, transporter systems, regulatory networks, and secondary-metabolite chemistries. Intriguingly, these organisms embody a remarkable duality: the mechanisms that empower them for efficient lignocellulose deconstruction underpin both the deterioration of living trees and wood products and at the same time a growing portfolio of applications in biotechnology, biomaterials, environmental remediation, and the circular bioeconomy.

Despite decades of research, important gaps remain. Current classifications into white, brown, and soft rot fungi are increasingly challenged by genomic and ecological evidence, highlighting the need for a more mechanistic understanding of decay strategies. Many key components of fungal wood utilization remain poorly characterized, including nutrient transporters, transcriptional regulators in Basidiomycota, and the ecological functions of secondary metabolites during wood colonization and competition. Likewise, links between genome content and actual decay behavior are still insufficiently resolved, particularly across diverse environmental conditions and developmental stages.

Future research should therefore move beyond inventories of genes and species and towards integrated studies that connect fungal morphology, physiology, ecology, and functional genomics. Such efforts will improve our ability to predict and manage fungal threats in forests and the built environment while simultaneously unlocking new opportunities for sustainable materials, bioprocesses, and bio-based products. Ultimately, a deeper understanding of the biology of wood-degrading fungi will be essential for harnessing their tremendous benefits while mitigating their negative impacts in a rapidly changing world.

## Data Availability

The datasets generated during and/or analyzed during the current study are available from the corresponding author on reasonable request.
